# Patterns of longitudinal subcortical atrophy over one year in amnestic mild cognitive impairment and its impact on cognitive performance: a preliminary study

**DOI:** 10.55730/1300-0144.5826

**Published:** 2024-03-11

**Authors:** Berrin ÇAVUŞOĞLU, Duygu HÜNERLİ, Derya Durusu EMEK SAVAŞ, Görsev YENER, Emel ADA

**Affiliations:** 1 Department of Medical Physics, Institute of Health Sciences, Dokuz Eylül University, İzmir, Turkiye; 2 Department of Neuroscience, Institute of Health Sciences, Dokuz Eylül University, İzmir, Turkiye; 3 Department of Psychology, Faculty of Letters, Dokuz Eylül University, İzmir, Turkiye; 4 Faculty of Medicine, İzmir University of Economics, İzmir, Turkiye; 5 İzmir International Biomedicine and Genome Institute, İzmir, Turkiye; 6 Department of Radiology, Faculty of Medicine, Dokuz Eylül University, İzmir, Turkiye

**Keywords:** Mild cognitive impairment, subcortical structures, magnetic resonance imaging, atrophy, neurocognitive functions

## Abstract

**Background/aim:**

Amnestic mild cognitive impairment (aMCI) is a risk factor for dementia, and thus, it is of interest to enlighten specific brain atrophy patterns in aMCI patients. We aim to define the longitudinal atrophy pattern in subcortical structures and its effect on cognition in patients with aMCI.

**Materials and methods:**

Twenty patients with aMCI and 20 demographically matched healthy controls with baseline and longitudinal structural magnetic resonance imaging scans and neuropsychological assessments were studied. The algorithm FIRST (FMRIB’s integrated registration and segmentation tool) was used to obtain volumes of subcortical structures (thalamus, putamen, caudate nucleus, nucleus accumbens, globus pallidus, hippocampus, and amygdala). Correlations between volumes and cognitive performance were assessed.

**Results:**

Compared with healthy controls, aMCI demonstrated subcortical atrophies in the hippocampus (p = 0.001), nucleus accumbens (p = 0.003), and thalamus (p = 0.003) at baseline. Significant associations were found for the baseline volumes of the thalamus, nucleus accumbens, and hippocampus with memory, the thalamus with visuospatial skills.

**Conclusion:**

aMCI demonstrated subcortical atrophies associated with cognitive deficits. The thalamus, nucleus accumbens, and hippocampus may provide additional diagnostic information for aMCI.

## 1. Introduction

Mild cognitive impairment (MCI) represents a transitional phase between dementia and normal aging [1]. Patients with MCI show dysfunction of cognitive domains involving memory, executive function, attention, language, and visuospatial, but do not fulfill the criteria for dementia [2]. Amnestic MCI (aMCI) that those with decreased memory ability have a greater risk of progression towards Alzheimer’s disease (AD) dementia [3]. Therefore, in vivo imaging biomarkers, which allow early diagnosis of patients with aMCI at high risk of developing AD dementia, are of prognostic importance.

Distinguishing the signs of MCI on magnetic resonance imaging (MRI) from normal age-related changes represents a challenge in brain imaging studies. MRI-based estimates of brain atrophy have been evidenced as a valid marker of neurodegenerative changes related to AD [4]. Being able to detect disease-related volumetric alterations before cognitive deterioration occurs is important for the early diagnosis of the disease. While most studies have focused primarily on the hippocampus and the neocortex because of their obvious role in memory processes and cognitive functions [5,6], subcortical structures have attracted much less attention [5,7]. Previous studies have revealed that patients with aMCI present the atrophy of subcortical grey matter (GM) including the thalamus, hippocampus, and other subcortical structures [8–10]. However, it still is unclear to what extent the aMCI affects the neurocognitive functions and subcortical structures. These structures might be differentially affected by long-term structural alterations and these changes may contribute to the cognitive deficit in aMCI patients.

MRI brain atrophy rate measured using serial images is a promising biomarker of AD progression [11,12]. Longitudinal volumetric analysis of subcortical structures might supply beneficial knowledge about the pattern of structural alterations that are associated with cognitive decline in aMCI. It is crucial to understand how the atrophy rate is related to cognitive decline to provide more effective plans for clinical research that can slow or prevent disease progression.

To the best of our knowledge, no study to date has examined the longitudinal progression of subcortical atrophy and its effect on cognition in aMCI. In the present study, we aimed to investigate the progression of subcortical atrophy over one year between aMCI and normal aging controls. We hypothesized that longitudinal subcortical GM atrophy contributes to an aMCI-related cognitive impairment with disease-specific patterns and that the subcortical GM atrophy pattern is associated with poorer cognitive test scores. We also hypothesized aMCI will have a greater subcortical GM atrophy rate and differ from normal aging controls.

## 2. Materials and methods

### 2.1. Participants

The retrospective study included 20 individuals with aMCI, and 20 age-, sex- and education-matched healthy elderly participants. Individuals with aMCI were recruited from the outpatient memory clinic of Dokuz Eylül University whereas healthy elderly participants were recruited from various community sources. Each subject underwent both baselines and repeat MRI scans and neuropsychological assessments at a one-year (14.85 ± 6.47 months) interval between 2013 and 2016.

For healthy elderly participants, the inclusion criteria were as follows: 1) no history of neurological abnormality and/or cognitive deficits (mini-mental state examination (MMSE) score ≥ 27); and 2) no self-reported cognitive complaints. The diagnosis of aMCI was performed according to NIA-AA diagnostic criteria [13]. In patients where amyloid-beta biomarkers were not evaluated according to the criteria, individuals whose neuronal damage occurring in MCI due to possible AD was detected with structural MRI of the temporal, parietal, hippocampus, and posterior cingulate [13]. The “positivity” was determined by the expert neurologists responsible for releasing the clinical diagnosis to the patients. The other clinical inclusion criteria of the aMCI patients were as follows: 1) memory complaints by the patient; 2) clinical dementia rating score of 0.5 (CDR [14]); 3) preserved daily life functionality; and 4) memory impairment defined with performances ≥ 1.5 standard deviation below for age- and education-matched controls in a battery of neuropsychological tests.

The clinical exclusion criteria of the aMCI patients were as follows: 1) systematic use of antidepressant drugs with anticholinergic side effects; and 2) actual participation in a clinical trial using disease-modifying drugs. For all participants, the exclusion criteria were as follows: 1) history of psychiatric and/or neurological including evidence of depression as demonstrated by Yesavage geriatric depression scale scores higher than 13; 2) history of alcohol and/or drug misuse and severe head injury; 3) presence of nonstabilized medical illnesses; 4) presence of a brain tumor, hydrocephalus, or vascular brain lesions; and 5) chronic use of narcotics, neuroleptics, analgesics, sedatives, or hypnotics and/or cognitive enhancers including acetylcholinesterase inhibitors.

For the determination of sample size, we used G*power (version 3.1) [15]. Since no study so far examined the longitudinal progression of subcortical atrophy in aMCI, we opted for the detection of at least hippocampal atrophy rate between groups according to a previous study [16]. We used the following criteria to calculate sample size: ANCOVA model, α = 0.05, 1–β = 0.80, number of groups = 2, number of covariates = 4. This calculation rendered a total sample size of 44. For the present study, we only achieved a total sample size of 40 due to the following exclusion criteria for each subject; poor image quality, MRI interval greater than one year, or lack of follow-up scanning.

### 2.2. Neuropsychological assessment and diagnostic criteria

A detailed neuropsychological test battery was applied to all participants. Global cognitive status was evaluated with MMSE [17]. In order to constitute cognitive domains, neuropsychological scores were first converted into z-scores according to age and education-adjusted norms. Then, composite scores were formed for each domain using the average z-scores of related neuropsychological tests. Memory domain was evaluated with Öktem Verbal Memory Processes Test (ÖVMPT) [18], attention/executive functions with Stroop test [19], WMS-R digit span test, verbal fluency test (phonemic), language domain with Boston Naming Test [20] and verbal fluency test (categorical), visuospatial domain with clock drawing test [21], and simply copying tests.

### 2.3. MRI acquisition and preprocessing

Both baseline and repeat brain MRI acquisitions were performed at the Department of Radiology in Dokuz Eylül University using the same 1.5 T Philips Achieva scanner (Philips Medical Systems, Best, the Netherlands) and a standard imaging protocol. A high-resolution 3D T1-weighted magnetization prepared rapid gradient echo sequence (repetition time: 25 ms, echo time: 6 ms, matrix: 512, field of view: 230 mm, number of signal averages: 1, slice thickness: 1 mm) was obtained for volumetric analysis. Axial T2 weighted dual-echo images were acquired for radiological assessment.

**Figure 1:**
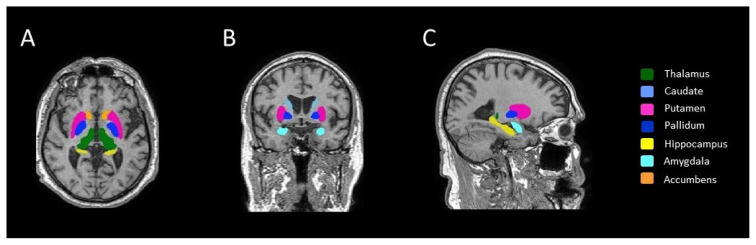
Example of automated segmentation of subcortical grey matter structure using FIRST on 3D T1-weighted images in A) axial, B) coronal, and C) sagittal planes.

Automatic volume estimation of the subcortical structures was performed by using the FSL software package^1^ . FMRIB’s integrated registration and segmentation tool (FIRST) [22] was applied to perform the segmentation and to estimate volumes in seven subcortical structures, including the thalamus, putamen, caudate nucleus, amygdala, globus pallidus, nucleus accumbens, and hippocampus, at each time point. FIRST initially performed an affine registration of 3D T1-weighted images to the MNI space. Next, the algorithm applies the inverse transformation to bring the images back to native space, followed by a boundary correction. The volume of each region was extracted bilaterally. All subcortical raw volumes were normalized for head size by multiplying by the volumetric scaling factor, which was automatically calculated using SIENAX (an adaptation of SIENA (Structural Image Evaluation, using Normalization, of Atrophy) for crosssectional measurement [23]. Finally, the left and right hemispheric measurements of the same structure were summed to reduce the number of comparisons between groups. An example of FSL-segmentation of subcortical structures for a healthy subject is demonstrated in Figure 1.

### 2.4. Statistical analysis

SPSS version 24.0 (IBM; Armonk, NY, USA) was used for all statistical analyses. Comparisons of demographic variables between groups were conducted using Mann–Whitney U test for continuous variables and the chi-square test for categorical variables. Change rates in subcortical volumetric measurements and z-scores of cognitive domains between two-time points were calculated with the following formula: [((data_follow-up_ −data_baseline_) / data_baseline_) / interval (in years)].

Group differences in the volumetric values and z-scores of cognitive domains were tested using analysis of covariance (ANCOVA). Age, sex, and education were included in the model as covariates of no interest, while baseline data served as additional covariates for comparing change rates between groups. Correlations between volume measurements and z-scores of cognitive domains were performed using either Pearson or Spearman correlation analysis after the Kolmogorov–Smirnov test was run to evaluate whether the variables show normal distribution. We also conducted partial correlations corrected for age, sex, and education.

The results were corrected with the Bonferroni correction for multiple tests [24]. For cognitive domains, corrections were applied to the four tests, and the statistical significance threshold was set to p ≤ 0.0125 (α/4). For volumetric analysis, corrections were applied to the seven tests, and the statistical significance threshold was set to p ≤ 0.007 (α/7). For correlation analysis, corrections were applied to the 28 tests (seven subcortical structures × four cognitive domains), and the statistical significance threshold was set to p ≤ 0.0018 (α/28). We presented the unadjusted p values and only the p values that survived Bonferroni correction were reported as significant.

## 3. Results

### 3.1. Demographic and clinical variables characteristics of the participants

The demographic and clinical characteristics by the group are shown in Table 1. There were no differences in age, sex, education, hand dominance, or GDS between controls and aMCI. At baseline, aMCI presented significantly lower scores in visuospatial skills (p = 0.005), episodic memory (p < 0.001), attention/executive functions (p = 0.002), and language (p = 0.007) domains compared to controls. Additionally, aMCI showed poorer scores on MMSE than controls. There were no differences in change rates between the groups. Table 2 presents the mean composite scores by group.

### 3.2. Subcortical volumetric analysis

At baseline, subjects with aMCI had a significantly lower volume in the thalamus (p = 0.003), hippocampus (p = 0.001), and nucleus accumbens (p = 0.003) compared to controls. There were no other significant subcortical volume differences between the aMCI and the control group. We also observed a greater hippocampus atrophy rate (p = 0.009) between groups; however, this result did not survive Bonferroni correction for multiple comparisons. Table 3 presents the comparisons of baseline volumes and the change rates in volumes between baseline and follow-up.

### 3.3. Correlation between the change rates of subcortical volume and cognitive performance

Correlations were analyzed for the total sample as a whole, as well as separately for the aMCI group. Only the significant correlations that survived the Bonferroni correction were reported.

**Figure 2:**
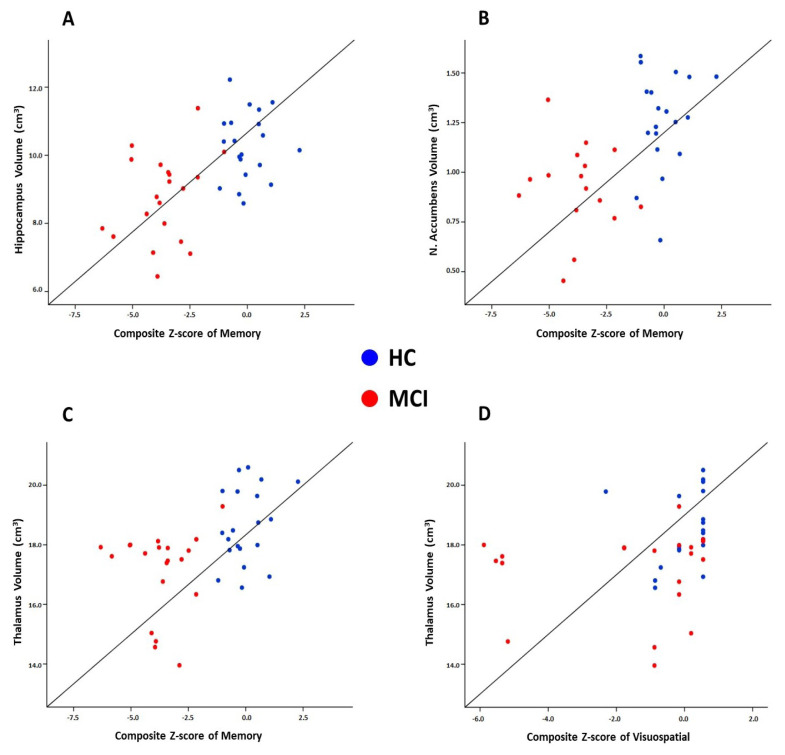
Scatter plots show the significant associations between subcortical volumes and composite scores of cognitive domains.

At baseline, strong correlations were detected between memory and hippocampus volume (r = 0.575, p < 0.001) (Figure 2A) and nucleus accumbens volume (r = 0.522, p = 0.001) (Figure 2B). Moreover, thalamus volumes were associated with memory (r = 0.483, p = 0.0016) (Figure 2C) and visuospatial skills (r = 0.506, p = 0.001) (Figure 2D). There were no significant findings when we used partial correlations corrected for age, sex, and education. No significant associations were found between change rates in subcortical volumes and cognitive tests. No significant correlations were observed in the aMCI group in terms of measures at baseline or change rate.

## 4. Discussion

In this study, we assessed the longitudinal pattern of structural alterations in subcortical deep GM structures and investigated their relations with neurocognitive functions in patients with aMCI. Compared with normal aging controls, we found that among all seven pairs of structures, the thalamus, nucleus accumbens, and hippocampus volumes were significantly lower in aMCI patients at baseline. In addition, we also showed associations between these structures and cognitive composite scores. These results suggested that the atrophy of these three subcortical structures was associated with clinical impairment in aMCI patients, which might be useful in the evaluation and diagnosis of aMCI.

At present, the roles of subcortical structures in neurodegenerative pathophysiology and its effect on cognitive outcomes in patients with aMCI remain poorly understood. Previous crosssectional imaging studies revealed varying degrees and significance of patterns of subcortical atrophy [8–10,25]. These findings indicate that there might be a specific subcortical atrophy pattern accompanied by a cognitive decline that can be early signs of aMCI. As expected, aMCI patients had significantly smaller hippocampal volumes in our study compared to cognitively normal subjects. We also showed a relation between hippocampal atrophy and memory decline, which is in line with previous studies. The hippocampus is involved in episodic and semantic memory, which are commonly impaired in patients with aMCI [26]. Numerous structural MRI studies have consistently demonstrated greater hippocampal atrophy in aMCI and hippocampal atrophy is typically seen as the earliest hallmark of AD [27–30].

The thalamus is a critical deep brain structure with interactions across cortical areas serving various cognitive processes [31]. Thalamic nuclei as a relay station regulate cortical network interactions and coordinate switching between networks [32]. In the current study, our findings were on par with previous studies indicating that the thalamus was atrophied in aMCI compared to controls [33,34], comparable to that of the hippocampus. The thalamic atrophy was suggested to be the result of amyloid accumulation and axonal degeneration [35]. The anterior nucleus of the thalamus, as well as the hippocampus, is one of the earliest locations of tau accumulation in the forebrain [32]. As part of the Papez circuit, the anterior thalamus is connected to the hippocampus and posterior cingulate, two key nodes of the default mode network (DMN), and it plays an important role in memory processing [32,36]. DMN, which supports episodic memory, has consistently been identified as dysfunctional in MCI [37]. Previous studies have also supported the relationship between thalamic atrophy and memory decline [38,39]. Moreover, we also found an association between the thalamus and visuospatial skills. The pulvinar nucleus is the posterior part of the thalamus and has strong connectivity with several areas of the visual cortex via its connections with the posterior parietal cortex [40]. Electrophysiology studies have provided evidence that pulvinar plays an important role in information transmission between visual cortical areas and visuospatial processing [41]. Taken together, the thalamus could be a possible subcortical neural substrate for cognitive impairment. Thus, thalamus atrophy may be used as another evaluation methodology for aMCI.

In line with the previous studies [7,8,25], the nucleus accumbens in our study were atrophied in aMCI and had an association with memory. The nucleus accumbens, located within the basal ganglia, can be affected in the early stages of AD and cause cognitive decline [42]. As a part of the limbic striatal loop, the nucleus accumbens contributes to memory and spatial learning with close connections with limbic structures of the prefrontal cortex, the amygdala, and the hippocampus [8]. The nucleus accumbens, the main component of the ventral striatum, receives a dense projection from the hippocampus and is involved in memory functions [43]. The previous study showed beta-amyloid deposition in the striatum in cognitively normal elders, as well as changes in the structure and functional connection between the striatum and other brain regions in MCI patients [44,45]. Neuroimaging studies reported that the nucleus accumbens had smaller volumes even in the preclinical stages of dementia compared to normal cognition [7,8,46], and they revealed that the nucleus accumbens atrophy was associated with an increased risk of progression to AD dementia in MCI patients [7]. These findings indicated that in addition to the thalamus and hippocampus, the atrophy in the nucleus accumbens may also play an important role in clinical impairment in aMCI.

Longitudinal assessments can reduce the impact of individual differences in neuroimaging evaluations. Previous studies revealed that the value of longitudinal measures of brain shrinkage is more informative than that of cross-sectional estimates [47,48]. Therefore, measurements of atrophy rates of subcortical structures may be a more practical and sensitive method to differentiate aMCI from controls. Moreover, there is a poor understanding of how longitudinal volume changes interact with cognitive performance. Atrophy rates were close between groups, in opposition to our hypothesis of greater atrophy rates in patients with aMCI than in controls. Contrary to the current study, several studies revealed a higher atrophy rate in the hippocampus [11,16,48–50] in aMCI patients than in the healthy group. Our negative findings may be due to the relatively small sample size of the aMCI group (n = 20) and short follow-up duration (one year), which was likely not long enough to catch statistically significant differences in atrophy rates. However, it should be noted that there are also studies that reported no significant differences between MCI in comparison with healthy elderly subjects [30,51]. Possible explanations for these inconsistent findings among previous studies could be the result of using the different segmentation techniques or statistical procedures used in each study. In our volume analysis, the group effect was controlled for age, sex, and education, and the multiple comparison correction with Bonferroni correction was also performed.

Although aMCI demonstrated clinically stable cognitive performance over one year, we found a trend for a greater hippocampal atrophy rate in aMCI patients (p = 0.009). This confirms our prediction of a higher atrophy rate being evident in aMCI subjects before they become aware of cognitive decline. The current findings suggest that monitoring longitudinal changes in hippocampal volume may differentiate age-related hippocampal damage from that associated with MCI. Hippocampal atrophy is not an AD-specific measure and is also present in patients with other neurodegenerative diseases such as hippocampal sclerosis, vascular dementia, and frontotemporal lobar degeneration, as well as in typical aging [52,53]. Therefore, hippocampal atrophy rate might be used as information in support of diagnostic evaluation for patients with aMCI. Furthermore, early detection of a greater hippocampal atrophy rate during healthy aging may help predict future MCI. It also provides a signal for future work in the development of biomarkers to clinically identify patients with aMCI.

The main strength of this study is the longitudinal design of a single cohort, which allows the examination of progression and clinical implications of subcortical GMV in patients with MCI. In addition, two groups were matched for age, sex, and educational level. Furthermore, all participants undertook a comprehensive neuropsychological assessment battery. Moreover, our findings were robust after applying multiple comparisons correction and considering the effects of age, sex, and education. To the best of our knowledge, the current study is the first investigation of the association between change rates in cognition and volumes of seven subcortical structures.

This study had some limitations. Patients who were not able to complete follow-up neurocognitive tests or MRI scanning led to decreased sample size. Due to our small sample size, our study may not be robust enough to determine the effect of longitudinal progression of subcortical atrophy in the aMCI group. Although this study’s sample size is relatively low, it is similar to published studies that include groups with small sample sizes [16,51]. Furthermore, the mean follow-up period of one year may not have been sufficient. Studies with longer follow-up periods have been capable of capturing volumetric change rates [16,48,50]. In addition, due to the short follow-up duration, it is difficult to make the final diagnosis for MCI patients, some of these patients may revert to normal cognition, while some may later progress to AD dementia. Previous longitudinal studies have reported no difference in MCI patients who do not convert to AD dementia compared to controls [30,51]. Therefore, it is plausible to assume that stable MCI patients in our patient group probably diminish the change rate differences between controls and MCI. The effect of cognitive impairment on volumetric changes in subcortical structures in the brain needs to be further investigated in a larger sample over an extended follow-up duration.

In conclusion, this study highlights the potential of subcortical GMV analysis to demonstrate the structural abnormalities accompanying MCI and its association with cognitive decline to obtain imaging-based measures for monitoring the disease. Our study presents valuable data as decreased volumes in the hippocampus, nucleus accumbens, and thalamus at baseline in aMCI patients. Also, we found that the baseline volumes of the thalamus, nucleus accumbens, and hippocampus had positive correlations with memory. Additionally, the thalamus was associated with visuospatial skills. Our results indicate that the atrophy of these structures in MCI may provide further information related to the clinical features. Since these measures were associated with neurocognitive performance at baseline, volume measures might be useful for investigating cognitive impairment. In addition, the present study explored a trend for a greater hippocampal atrophy rate in aMCI patients over one year. Our longitudinal findings suggest that prospectively following the hippocampal atrophy rate could be an important indicator for early detection of MCI. A prospective study with a longer observation duration will provide more information on its usefulness in the clinical practice of MCI.

## Figures and Tables

**Table 1:** Demographic and clinical characteristics of participants.

	Healthy controls (n = 20)	aMCI patients (n = 20)	p
**Age (years)**	70.15 ± 5.90	73.32 ± 4.63	0.052
**Sex (M/F)**	9/11	10/10	0.752
**Education (years)**	10.15 ± 5.21	9.48 ± 4.69	0.424
**Hand dominance (R/L/both)**	20/0/0	19/0/1	0.311
**MMSE**	29.26 ± 0.99	25.56 ± 3.52	**<0.001**
**Yesavage GDS**	5.75 ± 4.67	7.60 ± 5.34	0.236

M: male, F: female, R: right, L: left, MMSE: mini-mental state examination, Yesavage GDS: Yesavage geriatric depression scale. Significant p-value is highlighted in bold.

**Table 2:** Neuropsychological performances of participants.

	Healthy controls (n = 20)	aMCI patients (n = 20)	p
**Episodic memory**			
Baseline	0.00 ± 0.86	−3.68 ± 1.28	**< 0.001**
% change	0.00 ± 0.03	0.00 ± 0.00	0.845
**Attention/executive function**			
Baseline	0.05 ± 0.61	−0.85 ± 1.39	**0.002**
% change	0.00 ± 0.05	−0.01 ± 0.06	0.520
**Visuospatial**			
Baseline	0.03 ± 0.77	−1.60 ± 2.39	**0.005**
% change	−0.00 ± 0.02	0.00 ± 0.07	0.584
**Language**			
Baseline	−0.03 ± 0.78	−1.00 ± 1.50	**0.007**
% change	−0.00 ± 0.01	0.01 ± 0.16	0.583

Results of the ANCOVA model adjusted are reported. p values where the model is significant after Bonferroni correction for multiple comparisons are highlighted in bold.

**Table 3:** Volumetric measurements of baseline and follow-up MRI in MCI subjects compared to controls.

	Healthy controls (n=20)		aMCI (n=20)		p-value	
	Baseline volume	% change	Baseline volume	% change	Baseline volume	% change
**Thalamus**	18.6 ± 1.3	−0.3 ± 1.2	17.1 ± 1.4	−1.4 ± 2.4	**0.003**	0.079
**Caudate**	8.5 ± 0.9	−1.4 ± 2.7	8.6 ± 1.1	−1.9 ± 5.9	0.977	0.849
**Putamen**	12.1 ± 1.3	0.4 ± 4.7	10.8 ± 1.9	−1.7 ± 7.2	0.076	0.232
**Pallidum**	4.5 ± 0.5	0.6 ± 4.7	4.4 ± 0.8	−1.5 ± 7.0	0.299	0.384
**Hippocampus**	10.3 ± 1.0	−0.5 ± 4.2	8.8 ± 1.3	−5.0 ± 6.4	**0.001**	0.009
**Amygdala**	3.7 ± 0.6	−1.7 ± 10.4	3.2 ± 0.6	0.9 ± 11.2	0.016	0.924
**Accumbens**	1.3 ± 0.2	−7.3 ± 17.9	0.9 ± 0.2	−6.3 ± 21.7	**0.003**	0.418

Total volumes (mean ± standard deviation) are reported in cm_3_. Results of the ANCOVA model are reported. p values where the model is significant after Bonferroni correction for multiple comparisons are highlighted in bold.

## Footnotes

1 FMRIB (2022). FMRIB Software Library, version 6.0 [online]. Website www.fmrib.ox.ac.uk/fsl [accessed 05 January 2022].
